# Genome-Wide Analysis of Long Noncoding RNA Profiles in Seneca Valley Virus–Infected PK15 Cells

**DOI:** 10.3389/fvets.2022.825150

**Published:** 2022-03-03

**Authors:** Jianguo Dong, Dan Rao, Mingrui Chen, Pandeng Zhao, Li Huang

**Affiliations:** ^1^School of Animal Husbandry and Medical Engineering, Xinyang Agriculture and Forestry University, Xinyang, China; ^2^College of Animal Medicine, Henan University of Animal Husbandry and Economy, Zhengzhou, China

**Keywords:** SVV, genome-wide analysis, lncRNAs, mRNAs, signaling pathway

## Abstract

Long noncoding RNAs (lncRNAs) have been demonstrated to play key roles in various biological processes. However, the contributions of lncRNAs to Seneca Valley virus (SVV) infection and host defense remain largely unknown. In this study, differentially expressed lncRNAs and mRNAs in SVV-infected PK15 cells were detected by genome-wide analysis. A total of 14,127 lncRNAs and 63,562 mRNAs were identified, and 1,780 lncRNAs were differentially expressed. The functional prediction of SVV-induced lncRNAs showed high associations with biological regulation and many immunity-related signaling pathways, including the B-cell receptor pathway, RIG-I-like receptor signaling pathway, and NF-kappa B (NF-κB) signaling pathway. We next screened lncRNAs and target genes related to immune response pathways and further demonstrated their differential expression in SVV-infected PK15 cells. Our study investigated the function of lncRNAs involved in SVV infection and provided new insight into the pathogenic mechanisms of SVV.

## Introduction

Seneca Valley virus (SVV) is a nonenveloped, single-stranded RNA virus with a genome length of 7.3 kb that belongs to the genus *Senecavirus* in the family Picornaviridae and causes a disease characterized by vesicles, coronary band hyperemia, and lameness (1). The virus is indistinguishable from other swine vesicular diseases, such as foot-and-mouth disease (FMD), swine vesicular disease (SVD), vesicular stomatitis (vs.), and vesicular exanthema of swine (VES), which was first isolated from cell culture media and considered a cell culture contaminant in the USA in 2002. Subsequently, and recently, many countries have reported SVV infection associated with porcine idiopathic vesicular disease (PIVD), and its outbreak affects the productivity and economics of the pork industry to some extent (2–4).

Since the first SVV was isolated from swine in Guangdong Province, China, in 2015 (2), researchers have paid great attention to monitoring its prevalence and clarifying the clinical characteristics of different district-isolated strains. From 2015 to 2019, many provinces in China reported SVV infection in swine herds (5–7). Further understanding of the mechanism is crucial for the prevention and control of SVV-induced disease.

Long noncoding RNAs (lncRNAs), which are 200 bp to 100 kb in length, are highly conserved sequences of noncoding RNAs that usually reside in the nucleus (8). lncRNAs can regulate gene expression by transcriptional and posttranscriptional regulation and recruit transcription factors (9). Studies have indicated that lncRNAs play a role in immune processes, inflammatory responses, cancer, neurobiology, and stress. Meanwhile, lncRNAs can also regulate cell proliferation and death (10–12). Recent results have shown that lncRNAs play a vital role in regulating the transcription of viral and host genes, affecting the host antiviral response (13). lncRNAs have been demonstrated to regulate signaling steps and activation processes and can alter receptor function or regulate transcription factor binding and translocation. Some lncRNAs can directly modulate the expression of cytokines (14).

In this study, SVV-infected PK15 cells and uninfected cells were researched by high-throughput RNA sequencing. The differentially expressed (DE) mRNAs and lncRNAs were screened. The possible lncRNAs involved in SVV infection were screened.

## Materials and Methods

### Sample Preparation

K15 cells were cultured at 37°C and 5% CO_2_ in the high-glucose Dulbecco's modified Eagle medium (Gibco, CA, USA) containing 10% FBS (Gibco). PK-15 cells were infected with SVV CHhb17 (GenBank accession no. MG983756) at a multiplicity of infection (MOI) of 0.01 for 16 h. When PK15 cells showed an obvious cytopathic effect (CPE), the supernatant was discarded, and the cells were washed with phosphate-buffered saline three times. Then the cells were digested, and total RNA was extracted from the infected cells using an RNeasy Kit (Qiagen) according to the instructions of the manufacturer. The concentration and purity of RNA were measured by a Nanodrop (IMPLEN, CA, USA). RNA sample quality testing was completed by the company (RiboBio, Guangzhou, China).

### RNA Sequencing and Data Analysis

Three replicates of virus-infected and control samples were used for lncRNA sequencing (RNA-seq). Sequencing libraries of lncRNAs were constructed by the NEBNext Ultra Directional RNA Library Prep Kit (New England Biolabs, MA, USA) for Illumina HiSeq after the depletion of rRNA by Ribo-Zero™ rRNA Removal Kits (Epicenter, WI, USA). RNA-seq was performed using an Illumina HiSeq™2500 (Illumina, CA, USA). All the reads from RNA-seq were aligned to the pig genome using the TopHat 2.0 program (15), and the resulting alignment files were reconstructed with Cufflinks (16). CPAT was used to predict lncRNAs (17). According to the parameters of read length ≥ 200 nucleotides, exon number ≥ 2, FPKM ≥ 0.5, and coding potential score <0, the potential lncRNA transcripts were screened and identified using Potential Calculator, Pfam-scan, and Coding-Non-Coding Index. FPKMs (expected number of fragments per kilobase of transcript sequence per million base pairs sequenced) of both lncRNAs and mRNAs were calculated. When the *p* < 0.05, the DE transcripts were significantly different.

### Gene Ontology and KEGG Pathway Analyses

The GOseq method was used to conduct Gene Ontology (GO) analysis, and the genes were annotated cellular component (CC), biological process (BP), and molecular function (MF). KOBAS v3.0 software was used to analyze the KEGG pathways. *p* < 0.05 were considered significantly different.

### Real-Time PCR and Statistical Analysis

To further verify the sequencing results, PK15 cells were cultured at 37°C and 5% CO_2_ in high-glucose Dulbecco's modified Eagle medium containing 10% FBS. PK-15 cells were infected with SVV CHhb17 at a multiplicity of infection (MOI) of 0.01 for 16 h. Then the supernatant was discarded, the cells were washed with phosphate-buffered saline three times and digested, and total RNA was extracted from infected cells using an RNeasy Kit (Qiagen) according to the instructions of the manufacturer. First-strand cDNA was synthesized using Random Primer. The target genes were determined using an Applied Biosystems 7500 Real-Time PCR System. The relative expression levels of lncRNAs and the predicted target gene were normalized to GAPDH using the 2^−Δ*ΔCt*^ method. Data from three independent experiments were analyzed using one-way ANOVA. All data are demonstrated as the mean ± S.D. (^*^p < 0.05, ^**^p < 0.01, and ^***^p < 0.001).

## Results

### Differential Expression of Long Noncoding RNAs and MRNAs in SVV-Infected and Mock-Infected PK15 Cells

**Figure 1:**
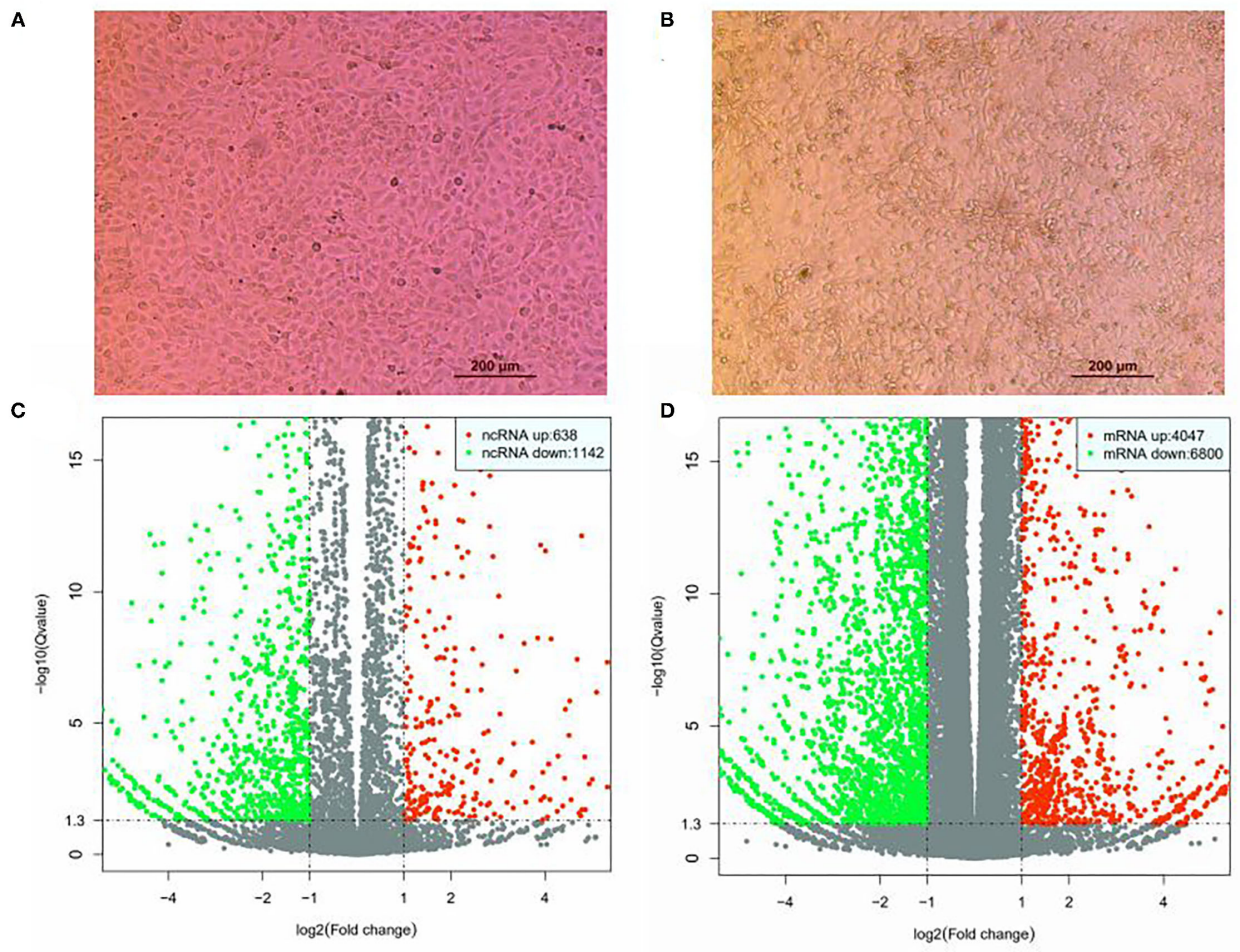
Differential expression of lncRNAs and mRNAs in SVV-infected and mock control PK15. **(A)** PK15 cells uninfected with SVV. **(B)** PK15 cells infected with SVV. **(C)** Volcano plots of DE lncRNAs. **(D)** Volcano plots of DE mRNAs.

PK15 cells cultured at 37°C and 5% CO_2_ were infected with SVV CHhb17 at a multiplicity of infection (MOI) of 0.01. At 16 h postinfection, the SVV-infected PK15 cells showed obvious shrinkage and clustering (Figure 1A). Before the disintegration and death of PK15 cells, the cells were digested and collected, and total RNA was extracted from the infected cells. The expression profiles of lncRNAs and mRNAs in the mock-infected and SVV-infected PK15 cells were analyzed by hierarchical clustering. A total of 14,127 lncRNAs and 63,562 mRNAs were identified. 8,287 lncRNAs and 54,445 mRNAs were expressed. As shown in Figure 1B, 1,780 lncRNAs were differentially expressed, including 638 upregulated lncRNAs and 1,142 downregulated lncRNAs. A total of 10,847 mRNAs were differentially expressed; of these mRNAs, 4,047 mRNAs were upregulated, and 6,800 mRNAs were downregulated (Figures 1C,D).

### Genomic Features of Long Noncoding RNAs in PK15 Cells

**Figure 2:**
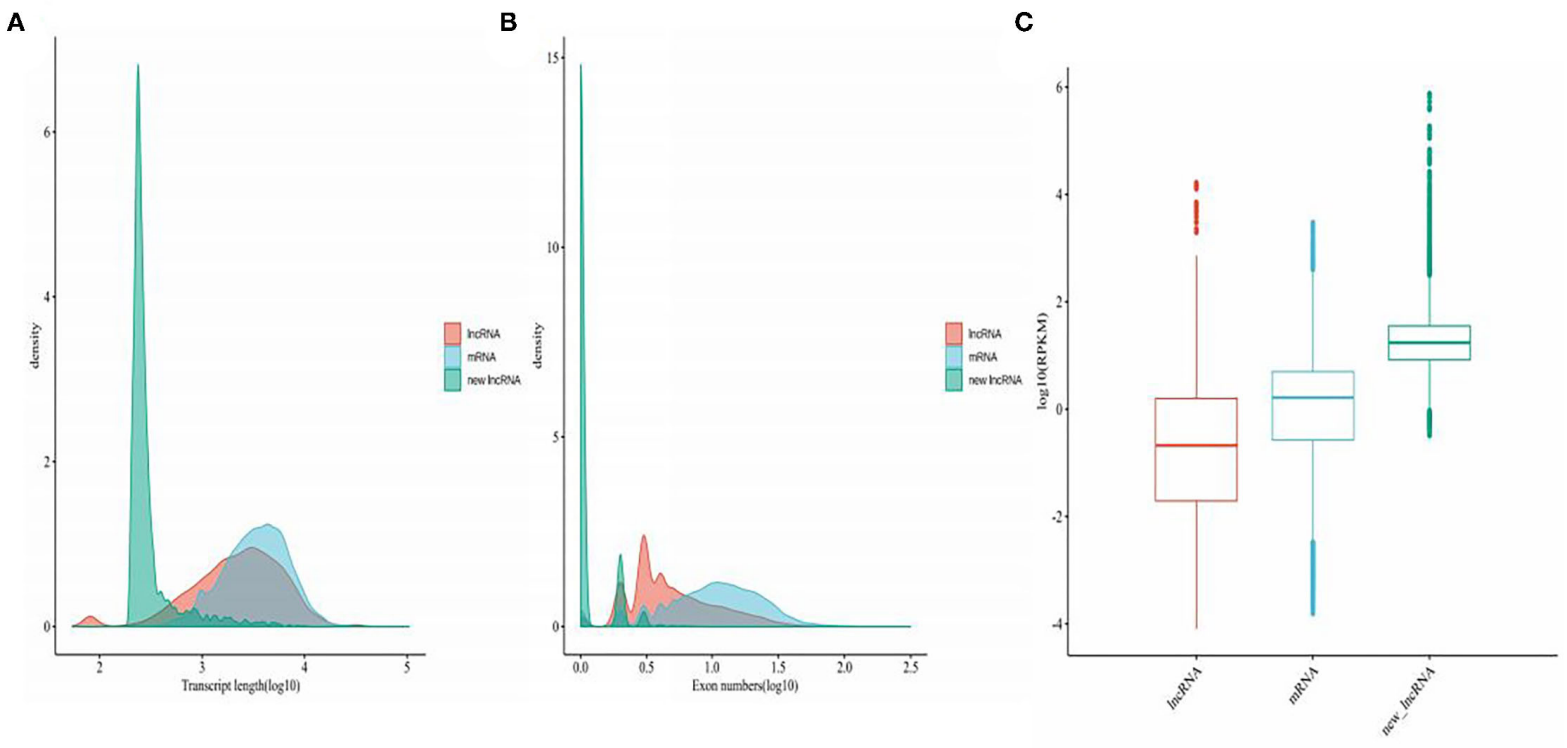
Genetic features of mRNAs, annotated lncRNAs, and novel lncRNAs. **(A)** Density map of the length distribution of mRNAs, annotated lncRNAs, and novel lncRNAs identified by RNA-seq analysis. **(B)** Distribution of the number of exons in mRNAs, annotated lncRNAs, and novel lncRNAs identified by RNA-seq. **(C)** Boxplot analysis of the expression features of mRNAs, annotated lncRNAs, and novel lncRNAs.

The genomic features of annotated lncRNAs, mRNAs, and new lncRNAs were analyzed. As shown in Figure 2A, new lncRNAs were shortest in transcript length. Compared with the transcript length of mRNAs, annotated lncRNAs were much shorter. The exons of annotated lncRNAs, mRNAs, and new lncRNAs were significantly different. The numbers of exons in new lncRNAs were fewest, and mRNAs possessed the most exons (Figure 2B). To further analyze the average expression levels of annotated lncRNAs, mRNAs, and new lncRNAs, boxplots were generated. As shown in Figure 2C, the expression levels of annotated lncRNAs were lowest and those of new lncRNAs were highest.

### Functional Prediction of Seneca Valley Virus–Induced Long Noncoding RNAs

**Figure 3:**
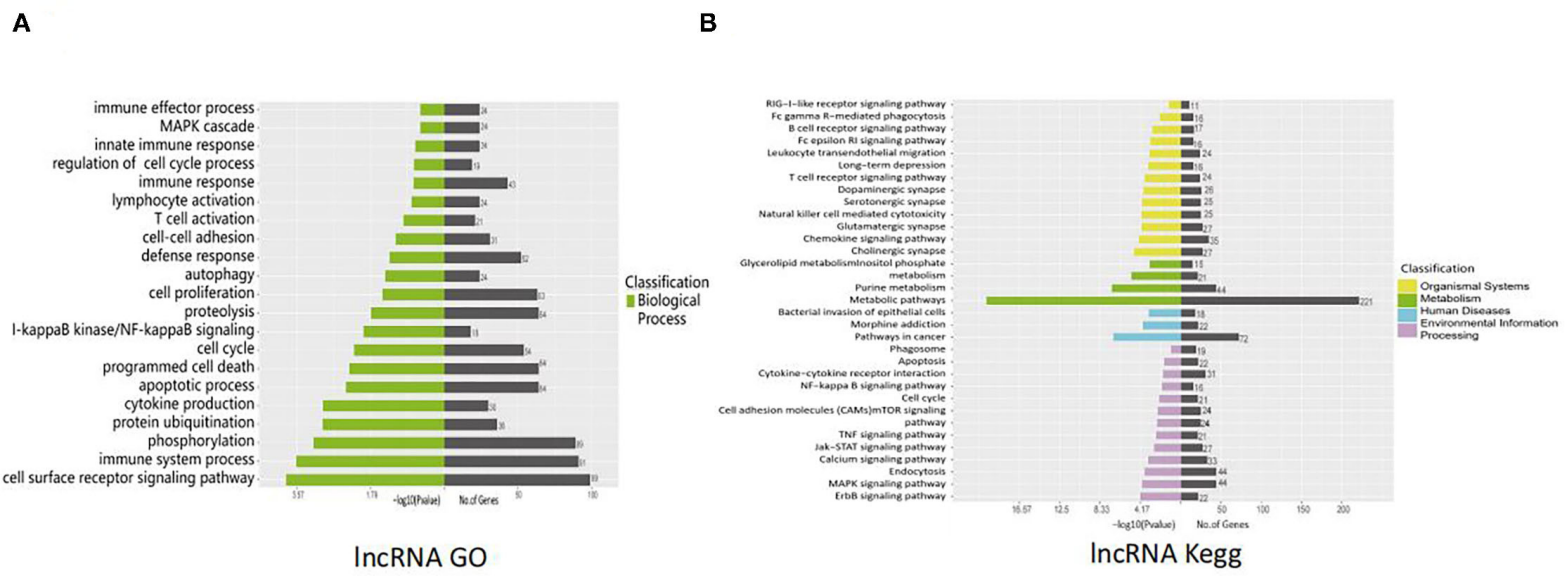
Functional prediction of SVV-induced annotated lncRNAs. **(A)** Gene Ontology (GO) analysis of the functional annotation of differentially expressed annotated lncRNAs. **(B)** KEGG pathway analysis of the functional annotation of differentially expressed annotated lncRNAs.

**Figure 4:**
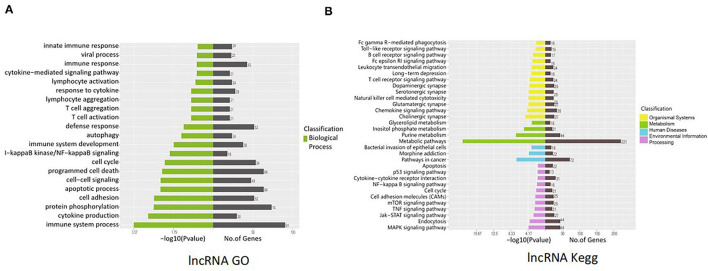
Functional prediction of SVV-induced new lncRNAs. **(A)** Gene Ontology (GO) analysis of the functional annotation of differentially expressed new lncRNAs. **(B)** KEGG pathway analysis of the functional annotation of differentially expressed new lncRNAs.

lncRNAs function through various mechanisms, and lncRNAs often regulate their neighboring genes. In our study, KEGG and GO enrichment analyses of the identified mRNAs were performed to investigate the function of these lncRNAs in SVV-infected PK15 cells. GO analysis showed that 21 pathways were possibly influenced by SVV infection (Figure 3A). KEGG analysis showed that these annotated lncRNAs were involved in the B/T-cell receptor signaling pathway, RIG-I-like receptor signaling pathway, cytokine–cytokine receptor interaction, NF-kappa B (NF-κB) signaling pathway, and Janus kinase (JAK)/signal transducer and activator of transcription (STAT) pathway, which are involved in immune and antiviral responses (Figure 3B). Furthermore, the function of new lncRNAs in SVV-infected cells was investigated. The results showed that most of the functions of these lncRNAs were related to immune cell differentiation and immune response (Figures 4A,B).

### Target Genes of Differentially Expressed Seneca Valley Virus–Induced LncRNAs

**Figure 5:**
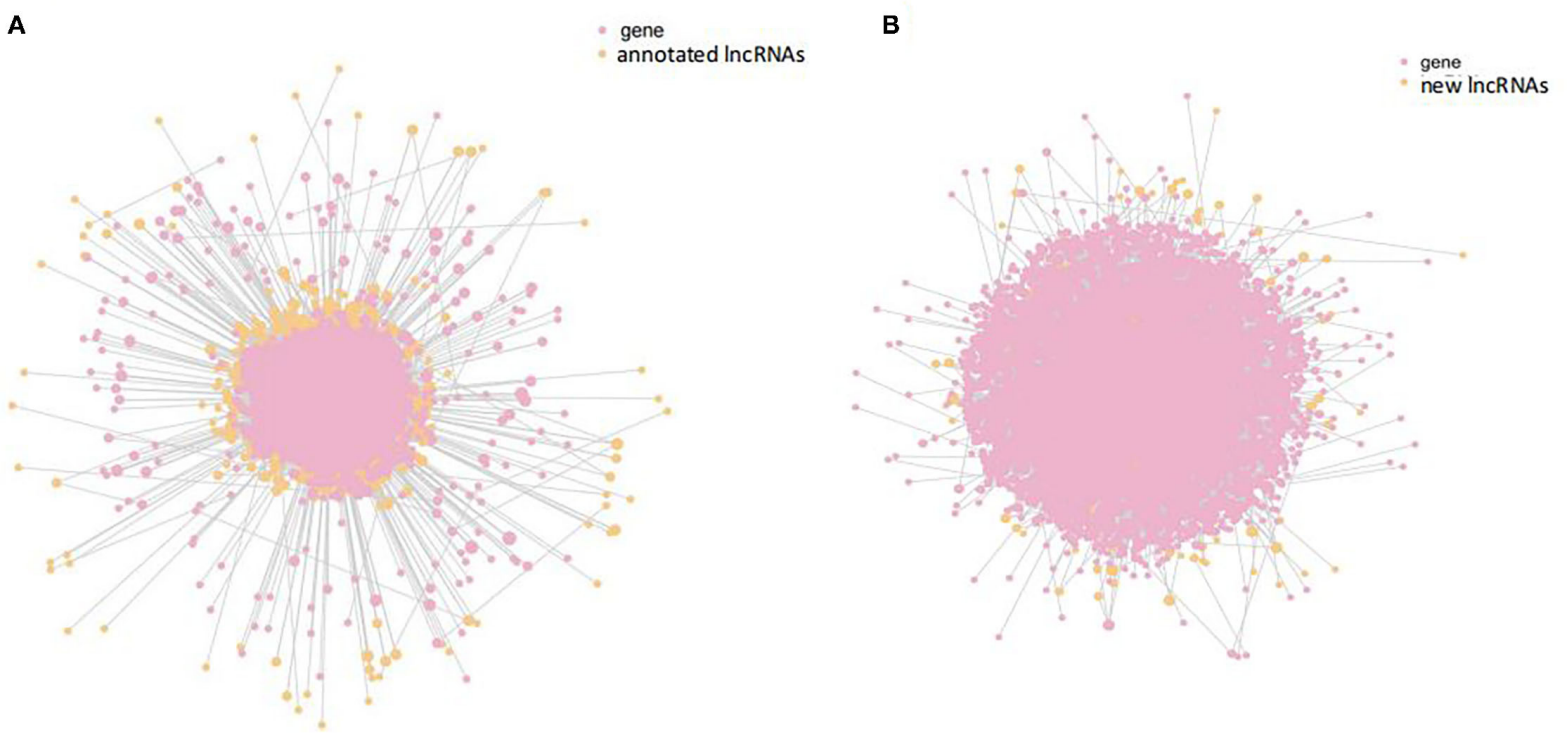
Interaction analysis of differentially expressed annotated lncRNAs and target genes. **(A)** Interaction analysis of differentially expressed annotated lncRNAs (orange) and target genes (pink). **(B)** Interaction analysis of differentially expressed new lncRNAs (orange) and target genes (pink).

Next, we analyzed the possible target genes of the DE lncRNAs. A total of 40,421 target genes might be regulated by DE-annotated lncRNAs, including 1,873 in cis and 38,548 in trans. The new DE lncRNAs might regulate 58,175 target genes, including 1,134 in cis and 57,051 in trans (Figures 5A,B). These genes are involved in organismal systems, metabolism, human diseases, environmental information, and host processing. Organismal systems contain Fc gamma R-mediated phagocytosis, Toll-like receptor signaling pathway, B-cell receptor signaling pathway, Fc epsilon RI signaling pathway, leukocyte transendothelial migration, long-term depression, T-cell receptor signaling pathway, dopaminergic synapse, serotonergic synapse, natural killer cell–mediated cytotoxicity, glutamatergic synapse, chemokine signaling pathway, and cholinergic synapse. Metabolism included glycerolipid metabolism and inositol phosphate metabolism. Human diseases included the bacterial invasion of epithelial cells, morphine addiction, and pathways in cancer. Processing included apoptosis, p53 signaling pathway, cytokine–cytokine receptor interaction, NF-kappa B signaling pathway, cell cycle, cell adhesion molecules (CAMs), mTOR signaling pathway, TNF signaling pathway, JAK-STAT signaling pathway, endocytosis, and MAPK signaling pathway.

### Validation of Differentially Expressed MRNA and Long Noncoding RNA Expression Levels

**Figure 6:**
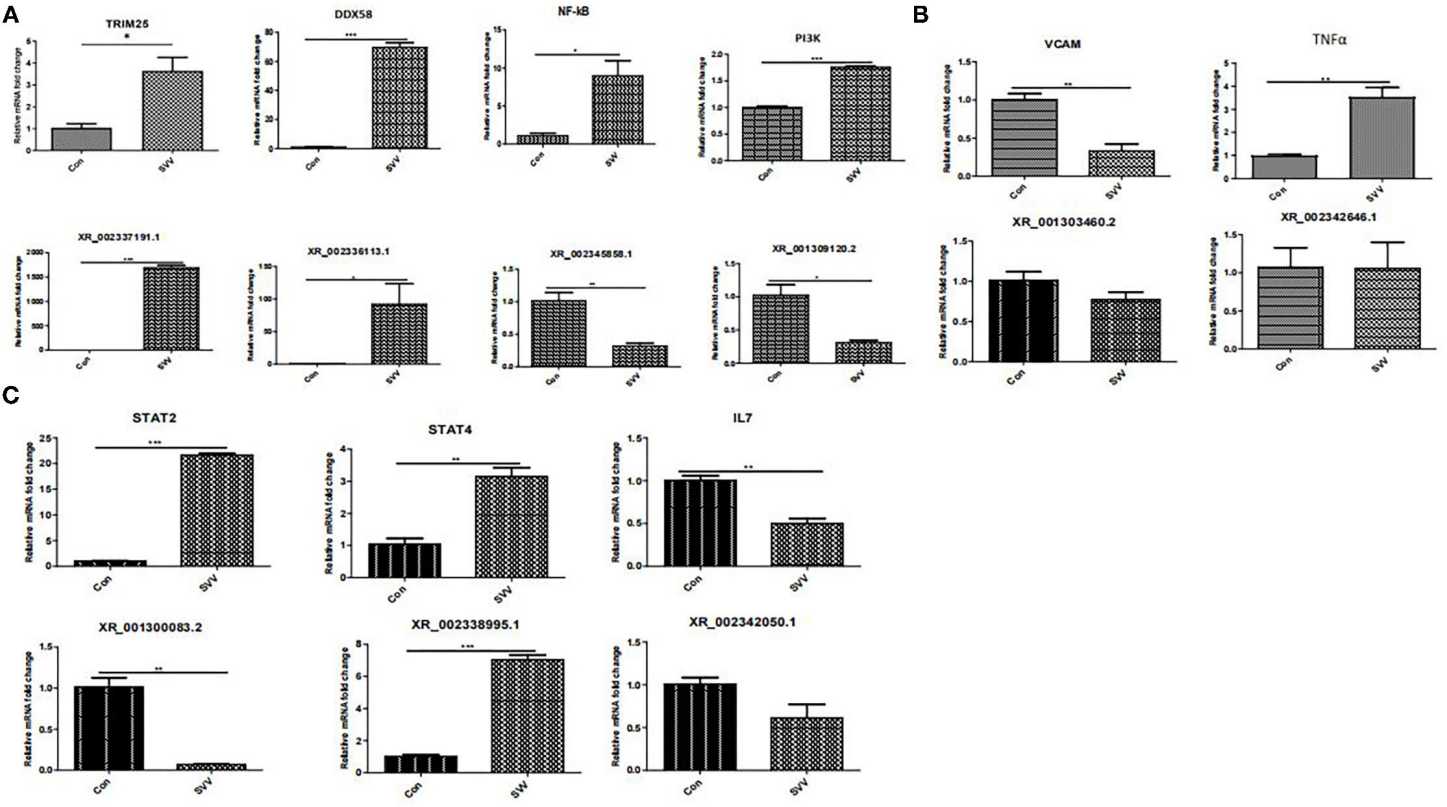
RT–qPCR confirmation of new lncRNAs and target genes in PK15 cells in response to SVV. **(A)** NF-kappa B signaling pathway. **(B)** TNF signaling pathway. **(C)** JAK-STAT signaling pathway. *Stands for significant difference, **stands for more significant difference, ***stands for most significant difference.

To confirm the results of RNA sequencing, several lncRNAs and target genes that may be involved in the immune response were verified by real-time RT–PCR. Several important signaling pathways, including the NF-kappa B signaling pathway, TNF-α signaling pathway, and JAK-STAT signaling pathway, which play an important role in the innate antiviral immune response, were assessed by qPCR. As shown in Figure 6, NF-κB, DDX58 (DExD/H-box helicase 58), TRIM25 (tripartite motif containing 25), PI3K (phosphoinositide 3-kinase), STAT2, STAT4, and TNF-α (tumor necrosis factor alpha) were upregulated in SVV-infected PK15 cells (Figures 6A–C), while VCAM (vascular cell adhesion molecule) and IL7 (interleukin 7) were downregulated (Figures 6B,C). Moreover, the associated lncRNAs, XR_002337191.1, XR_002336113.1, XR_002345858.1, XR_001300083.2, XR_002338995.1, XR_002342050.1, XR_001303460.2, XR_001309120.2, and XR_002337463.1, were differentially expressed in SVV-infected PK15 cells. All results were consistent with the RNA sequencing results, confirming the reliability of the sequencing data.

## Discussion

Viral and host interactions are an important aspect to explore the pathogenesis of viruses. With a deep understanding of lncRNAs, research has shown that the lncRNA expression is correlated with neighboring protein-coding genes (18, 19). By regulating lncRNA neighboring protein-coding genes, a large number of lncRNAs are produced in the host and their rapid transcription lncRNAs, and they can regulate the molecular biological function of the host to affect virus replication. Furthermore, lncRNAs can encode peptides that directly regulate host cell function (20), implying that more unknown functionality of lncRNAs needs to be further explored.

Although many studies indicate that lncRNAs regulate the evasion of immune responses of viruses (21, 22), research on lncRNAs in the field of antiviral therapy is still in its infancy. Recent research shows that lncRNAs are regulators of antiviral responses through the modulation of cytokine/IFN pathways or receptor function (14). Cytokines/IFNs are vital immune defense mechanisms that protect the host. The cytoplasmic receptors, melanoma differentiation–associated gene (MDA5), and retinoic acid–inducible gene I (RIG-I), are major receptors for detecting viral nucleic acids. Foreign RNAs in the cytosol are mainly recognized by RIG-I and MDA5 receptor activation and result in nuclear translocation of transcription factors such as NF-κB and interferon regulatory factors (IRFs), which induce the expression of proinflammatory cytokines and the production of type I IFN (IFN-I). IFN-I can interact with IFN receptors and activate the Janus kinase/signal transducers and activators of transcription (JAK-STAT) pathway to secrete antiviral proteins, playing antiviral functions. In our study, KEGG and GO analyses showed that these lncRNAs in SVV-infected PK15 cells were involved in the RIG-I-like receptor signaling pathway, and NF-κB, DDX58, and TRIM25 were upregulated in the SVV-infected PK15 cells.

TRIM25 is a type I and type II IFN-inducible E3 ligase that belongs to tripartite motif proteins. It was first identified as an “estrogen-responsive finger protein” (EFP) (23). TRIM25 is involved in many cellular processes, especially innate immunity (24). TRIM25 plays a role by mediating K63-linked polyubiquitination to regulate RIG-I signaling (25). Meanwhile, type I IFN production is related to TRIM25-mediated K48-linked ubiquitination and subsequent proteasomal degradation of the larger MAVS isoform (26). TRIM25 is involved in RIG-I/MDA5-mediated induction of IFN-I pathways, and studies have shown that TRIM25-mediated RIG-I CARD ubiquitination and RIG-I signal transduction are suppressed by influenza A virus NS1 (27, 28). A severe acute respiratory syndrome coronavirus nucleocapsid can interfere with TRIM25-mediated RIG-I ubiquitination to inhibit type I IFN production (29). The nucleocapsid protein of the porcine reproductive and respiratory syndrome virus also interferes with TRIM25-mediated RIG-I ubiquitination to antagonize its antiviral activity (30). In addition, TRIM25 regulates the viral proteins for ubiquitination and degradation to affect virus replication. TRIM25 can promote the ubiquitination and degradation of VP3 of an infectious bursal disease virus to inhibit virus replication (31). Recent research has shown that Lnczc3h7a promotes a TRIM25-mediated K63-linked ubiquitination of RIG-I and accelerates downstream signal transduction, affecting effective antiviral defense (32). Furthermore, the expression level of the annotated lncRNA, XR_002337191.1, which was predicted to target TRIM25, was upregulated in SVV-infected PK15 cells. Our results showed that the expression level of lncRNA, XR_002336113.1, and its predicted target gene, DDX58, were both upregulated in SVV-infected PK15 cells. DDX58 is a cytosolic viral RNA receptor and is involved in the RIG-I/MDA5 receptor signaling pathway to induce type I IFN-mediated host protective innate immunity against viral infection (33, 34).

Moreover, the expression level of the annotated lncRNA, XR_002345858.1, which was predicted to target NF-κB, was downregulated in SVV-infected PK15 cells; interestingly, NF-κB was upregulated after SVV infection. These results suggest that SVV infection might activate the RIG-I and NF-κB signaling pathways and that XR_002345858.1 might negatively regulate NF-κB reproduction. Researchers have demonstrated that the lncRNA, NKILA, regulates HIV-1 replication and latency by repressing NF-κB signaling (35). Whether XR_002345858.1 regulates SVV infection needs to be further explored.

lncRNAs not only regulate signaling steps and activation processes but also alter receptor function or regulate transcription factor binding and translocation. Cytokines such as IL6 and IL7 activate the JAK-STAT signaling pathway to initiate an antiviral response (36, 37). Our study found that many differentially expressed lncRNAs are involved in the JAK-STAT signaling pathway. Meanwhile, the STAT2 and STAT4 expression levels were significantly increased after SVV infection, together with those of the annotated lncRNA XR_002338995.1 target of STAT4. The lncRNA XR_002342050.1 and its predicted target gene IL7 were both downregulated in SVV-infected PK15 cells (Figure 6C). These results indicated that SVV infection can regulate the JAK-STAT signaling pathway. SVV regulates the expression of IL7 by the lncRNA XR_002342050.1-mediated regulatory mechanism and should be researched in the future. B cells are an important component of adaptive immunity (38). They produce and secrete millions of different antibody molecules to recognize a different foreign antigen. The B-cell receptor (BCR) is an integral membrane protein complex that recognizes antigens and activates downstream B-cell receptor signaling pathways. PI3K (phosphatidylinositol 3-kinase) is an important downstream effector of the BCR signaling pathway (39, 40). Meanwhile, PI3K also plays a role in multiple signaling pathways, such as JAK-STAT and NF-κB. Our study found that the expression of PI3K was increased by SVV infection, although the lncRNA XR-001309120.2 target of PI3K was downregulated in SVV-infected PK15 cells. TNF-α is a well-known pro-inflammatory factor that plays an important role in the immune response; it induces VACM1 and activates NF-κB, and a variety of noncoding RNAs participate in the regulation of TNF (41, 42). Our results showed that SVV infection upregulated the TNF-α and NF-κB expressions, and interestingly, VACM1 expression was inhibited. The mechanism by which SVV regulates VACM1 needs further study.

Our data have provided new insights into the interaction between the SVV and host response. We analyzed the expression changes of lncRNAs and targeted mRNAs in SVV-infected PK15 cells. The data suggested that some lncRNAs may be involved in the process of SVV infection and provided a new direction for understanding host lncRNA–mRNA interactions during SVV infection. The role of lncRNAs in the defense against SVV invasion will be explored further in future work.

## Data Availability Statement

The datasets presented in this study can be found in online repositories. The names of the repository/repositories and accession number(s) can be found in the article/supplementary material.

## Author Contributions

JD conceived and designed the experiments. JD, MC, and DR performed the experiments. JD, PZ, and LH analyzed the data and wrote the paper. All authors contributed to the article and approved the submitted version.

## Funding

This study was supported by the Youth Backbone Training Program of Colleges and Universities in Henan Province (Jiaogao [2020] No. 354), Young Teachers Scientific Research Fund Project of Xinyang Agriculture and Forestry University, Scientific and Technological Project of Henan Province (212102110364), Scientific and Technological Project of Henan Province (202102110095), Scientific and Technological Innovation Team Project of Xinyang Agriculture and Forestry University, and Key and Cultivation Discipline of Xinyang Agriculture and Forestry University (ZDXK201702).

## Conflict of Interest

The authors declare that the research was conducted in the absence of any commercial or financial relationships that could be construed as a potential conflict of interest.

## Publisher's Note

All claims expressed in this article are solely those of the authors and do not necessarily represent those of their affiliated organizations, or those of the publisher, the editors and the reviewers. Any product that may be evaluated in this article, or claim that may be made by its manufacturer, is not guaranteed or endorsed by the publisher.
